# In Vitro Effect of 3D Plates Used for Surgical Treatment of Condylar Fractures on Prostaglandin E_2_ (PGE_2_) and Thromboxane B_2_ (TXB_2_) Concentration in THP-1 Macrophages

**DOI:** 10.3390/ijms18122638

**Published:** 2017-12-08

**Authors:** Maciej Sikora, Marta Goschorska, Irena Baranowska-Bosiacka, Dariusz Chlubek

**Affiliations:** 1 Department of Maxillofacial Surgery, Hospital of the Ministry of Interior, Kielce, Wojska Polskiego 51, 25-375 Kielce, Poland; sikora-maciej@wp.pl; 2 Department of Biochemistry and Medical Chemistry, Pomeranian Medical University, Powst. Wlkp. 72; 70-111 Szczecin, Poland; irena.bosiacka@pum.edu.pl (I.B.-B.); dchlubek@pum.edu.pl (D.C.)

**Keywords:** 3D titanium mini-plates, condylar fracture, prostaglandin E_2_ (PGE_2_), thromboxane B2 (TXB2), macrophages, inflammation

## Abstract

Recent studies have shown promising results concerning the effectiveness of 3D plates in terms of stabilization of condylar fractures. Despite the use of new techniques and new materials, we can still observe certain side effects, including the immune reaction of the body, which may lead to the excessive inflammation. The aim of this paper was to determine how the production of prostaglandin E_2_ (PGE_2_) and thromboxane B_2_ (TXB_2_) in THP-1 monocytes/macrophages is influenced by the titanium 3D plates and dedicated screws. The experiments were conducted on THP-1 monocytic cell line and macrophages derived from a THP-1cells. The concentrations of PGE_2_ and TXB_2_ released were measured by using immunoassay kit. Verification of plate-induced activation of THP-1 monocytes and macrophages and initiation of inflammatory reaction was conducted by flow cytometry. Despite some differences in the content of the implant devices our results showed that these plates did not statistically significantly increase the production of these prostanoids. Osteosynthesis of condylar fractures using 3D titanium mini-plates seems to be a good alternative to traditional plates due to their lack of stimulating the cyclooxygenase-dependent production of prostanoids; limiting the development of inflammatory reactions.

## 1. Introduction

Stable osteosynthesis carried out with screwed miniplates is currently the most popular surgical method for treating condylar fractures [1,2]. However, its two major techniques, the single- and double-plate, are associated with major technical problems [3,4,5]. The single-plate technique may result in plate fracture or the loosening of screws, leading to secondary displacement. Double-plate osteosynthesis, often recommended to avoid these issues, increases the difficulty in the correct positioning of the plate due to the use of at least six screws on a small area [6].

The recently introduced 3D plates seem to be the solution to the aforementioned problems [7]. Their shape and the locations of the screws make it possible to use a single plate only, and their small size enables fixation on a very small area [8]. Various reports show promising results concerning their effectiveness in terms of stabilization of fractures, tolerance of the operated area to the applied forces, and plate resistance [9,10,11]. However, the selection of a 3D plate type should not only be decided by purely technical matters [12]. Before deciding which 3D plate is best suited for the osteosynthesis of condylar fractures, it seems necessary to determine their effect on the human body, including cellular, humoral and vascular mechanisms that constitute the inflammatory reaction [12].

Implantable devices are used on a large scale in the treatment of various diseases [13]. Despite the continuous introduction of new techniques and new materials, we can still observe certain side effects [14]. One of them is the immune reaction of the body, which may lead to the excessive inflammation. Chronic inflammation may substantially influence the lifetime of the implant in the body, in extreme cases necessitating the removal of an implant. Therefore, it seems crucial to establish how the implantation of the plate and screws induces the inflammation [13].

A number of reports show that the implantation of biomaterials may result in the release of cytokines, chemokines and the activation of the complement, which in turn affects the host-implant interaction [12]. For example, an inflammatory reaction results in the failure of 10–20% of titanium implants [15,16]. This is why in this paper we tried to determine how the available condylar 3D plates and their screws affect the body, in particular due to the lack of reports on their effect on the synthesis of prostanoids (PGE_2_ and TXB_2_), the mediators of the inflammatory state.

Inflammation is significantly associated with the activity of the macrophages, cells participating in the body’s response to various factors, including implants and biomedical devices [17]. Monocytes/macrophages are one of the first to come into contact with the implant immediately after grafting [18]. Their secretion of cytokines and growth factors initiates both reparative and destructive processes [12,19] The initial phase after implantation involves the intense secretion of pro-inflammatory cytokines such as interleukin 1β (IL-1β), interleukin IL-6 (IL-6), tumor necrosis factor alfa (TNF-α), and chemokines such as monocyte chemoattractant protein-1 (MCP-1) and macrophage inflammatory protein-1α (MIP-1α) [12,20,21].

Macrophages are also known to produce cyclooxygenase (COX), with COX-1 and COX-2 catalyzing the conversion of arachidonic acid (AA) into prostaglandin H_2_ (PGH_2_) [22,23]. The resultant PGH_2_ is a precursor for biologically active prostanoids, e.g., prostaglandin E_2_ (PGE_2_), thromboxane A_2_ (TXA_2_) and prostaglandin I_2_ (PGI_2_) [24]. Until quite recently it was believed that COX-1 is a constitutive enzyme and does not play a significant role in inflammation [25]. However, it is now known that in some tissues this enzyme plays a role in the initial phase of the response to factors initiating the synthesis of prostanoids, and in the resolution of inflammation [24,26]. COX-2 is an enzyme expressed in response to e.g., proinflammatory cytokines or cytokines produced by cell growth factors (including monocytes), and has a dominant role in chronic inflammation [26,27].

### Aim of the Study

The aim of this paper was to determine how the production of PGE_2_ and TXB_2_ in THP-1 monocytes/macrophages is influenced by the titanium 3D plates and dedicated screws used in the surgical treatment of condylar fractions.

## 2. Results

### 2.1. Plate-Induced Activation of THP-1 Monocytes and Macrophages

**Figure 1:**
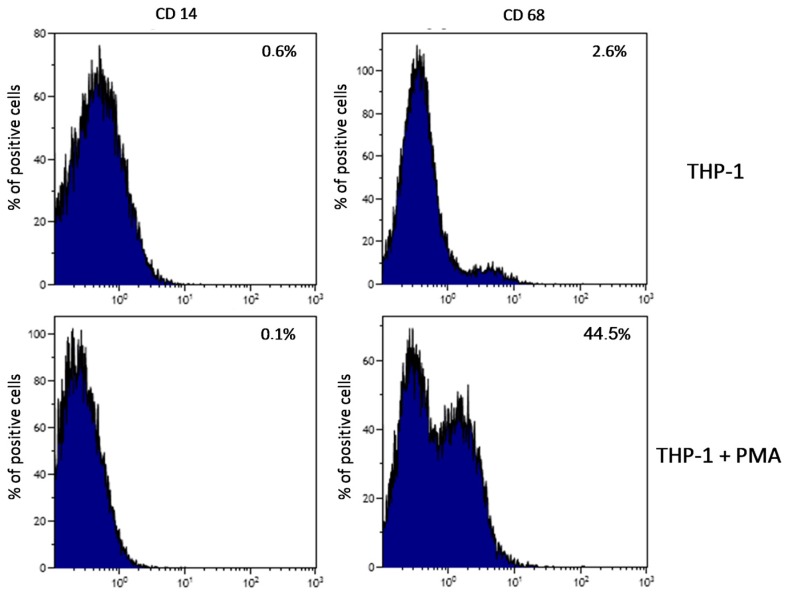
The effect of a plate on the activation of THP-1 monocytic cells. THP-1 monocytic cells were incubated in the presence of a plate without PMA (upper quadrants) and treated with PMA (lower quadrants). Expression was determined by flow cytometry. CD68 expression (marker for macrophage differentiation) increased after 48 h treatment with PMA (200 nM) as compared to non-treated cells. Expression of CD14 (marker for monocyte differentiation) did not change. Experiments were conducted as six separate assays, each assay in three replicates, (*n* = 18).

Plate-induced activation of THP-1 monocytes and macrophages, and initiation of inflammatory reaction showed that CD68 expression (marker for macrophages differentiation) increased after 48 h treatment with PMA (200 nM) as compared to non-treated cells. Expression of CD14 (marker for monocyte differentiation) did not change (Figure 1).

### 2.2. Prostaglandin E_2_ (PGE_2_) in THP-1 Monocytes

#### 2.2.1. Incubation of THP-1 Cells with SYNTES (S) Plates

**Figure 2:**
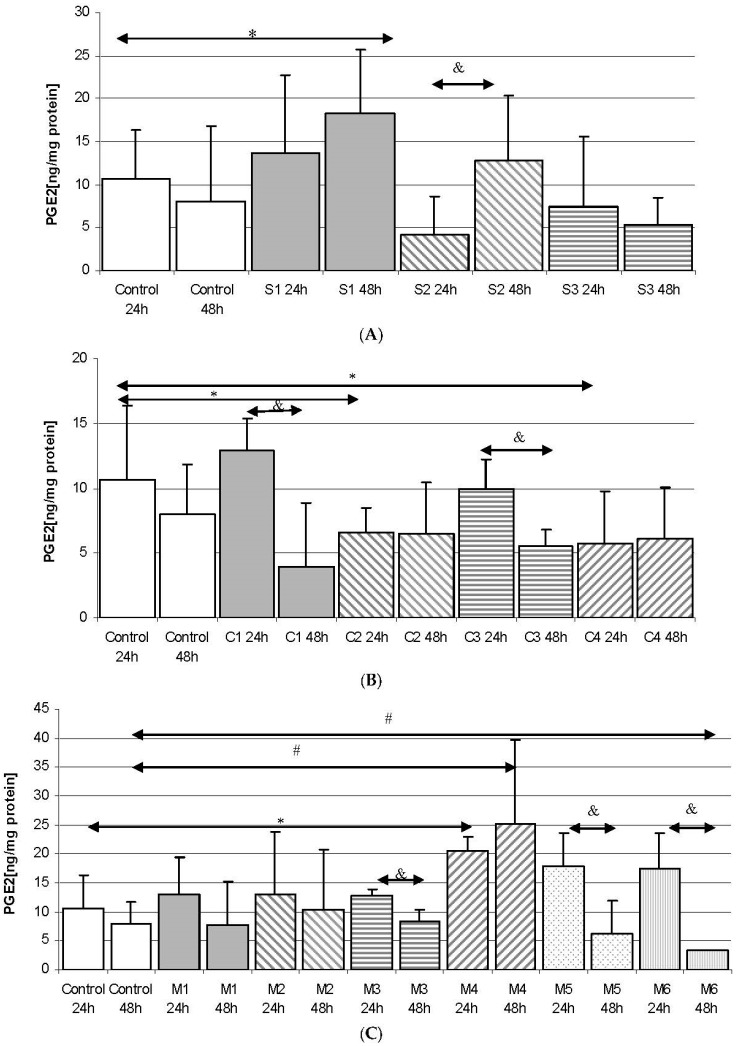
The concentration of prostaglandin E_2_ (PGE_2_) in THP-1 monocytes cultured with various plates and dedicated screws used for surgical treatment of patients with condylar fractures. THP-1 cells were cultured for 24 h and 48 h in RPMI medium with 10% FBS with various plates and dedicated screws: (**A**) SYNTES (S1, S2, S3); (**B**) MARTIN (C1, C2, C3, C4); (**C**) MEDARTIS (M1, M2, M3, M4, M5, M6), for details see Table 1. After incubation the cells were harvested by scraping and PGE_2_ concentration was measured by ELISA. Experiments were conducted as six separate assays, each assay in three replicates, (*n* = 18). * Statistically significant differences in comparison to control 24 h (*p* ≤ 0.05), # Statistically significant differences in comparison to control 48 h (*p* ≤ 0.05), & Statistically significant differences in comparison to appropriate plate 24 h (*p* ≤ 0.05). Control cells were incubated in RPMI medium with 10% FBS.

The incubation of THP-1 cells with SYNTES S1 plate for 48 h resulted in an increase in PGE_2_ concentration (by 128%) compared to Control 48, but not statistically significantly (*p* = 0.065). Incubation of cells with SYNTES S2 for 24 h resulted in a statistically significant decrease in PGE_2_ in the medium (by 60%) compared to Control 24 h (*p* = 0.033). Extending incubation time to 48 h (S2 48 h) resulted in a significant 200% increase in PGE_2_ in the medium relative to S2 24 h, but not statistically significantly with respect to Control 48 h (Figure 2A).

#### 2.2.2. Incubation of THP-1 Cells with MARTIN (C) Plates

Incubation of THP-1 monocytes for with the C2 plate for 24 h resulted in a statistically significant decrease in PGE_2_ concentration in the medium relative to cells incubated under control conditions (Control 24), by 38% at *p* = 0.021. Similarly, incubation of cells with C4 24 h showed a statistically significant decrease in the concentration of this prostaglandin by 47% relative to Control 24 h. Extending the incubation time from 24 h to 48 h resulted in a significant decrease in PGE_2_ in culture incubated with the C1 24 h plate compared to C1 48 h. Similar regularity was observed for C3 24 h cell incubation compared to C3 48 h (47% decrease, *p* = 0.03). Incubation of THP-1 monocytes with MARTIN plates for 48 h resulted in no statistically significant increase in PGE_2_ for any of the plates used (Figure 2B).

#### 2.2.3. Incubation of THP1 Cells with MEDARTIS (M) Plates

The incubation of THP-1 monocytes with MEDARTIS M4 plate for 24 h resulted in a statistically significant increase (by 91%) in PGE_2_ concentration in the medium relative to Control 24 (*p* = 0.045). Extending the incubation time resulted in a further significant increase in PGE_2_ compared to Control 48 h, by 213% (*p* = 0.035). Incubation with the M6 48 h plate resulted in a statistically significant decrease in PGE_2_ compared to Control 48 h, by 58% (*p* = 0.042). A statistically significant decrease in PGE_2_ concentration was also demonstrated for cells incubated for 24 h with M3, M5 and M6 plates (34%, *p* = 0.024; 66%, *p* = 0.031; 80%, *p* = 0.035, respectively) (Figure 2C).

### 2.3. Prostaglandin E_2_ (PGE_2_) in THP-1 Macrophages

Incubation time had a significant effect on PGE_2_ synthesis in macrophages in control cultures—prostaglandin concentration in Control 48 h was 54% higher than in Control 24 h (*p* = 0.020).

#### 2.3.1. Incubation of Macrophages with SYNTES Plates (S)

**Figure 3:**
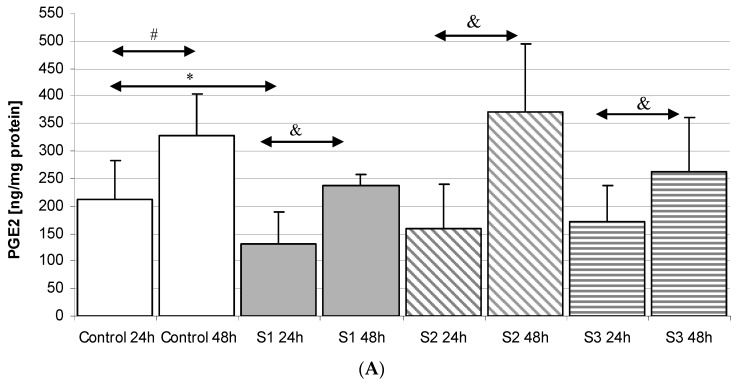

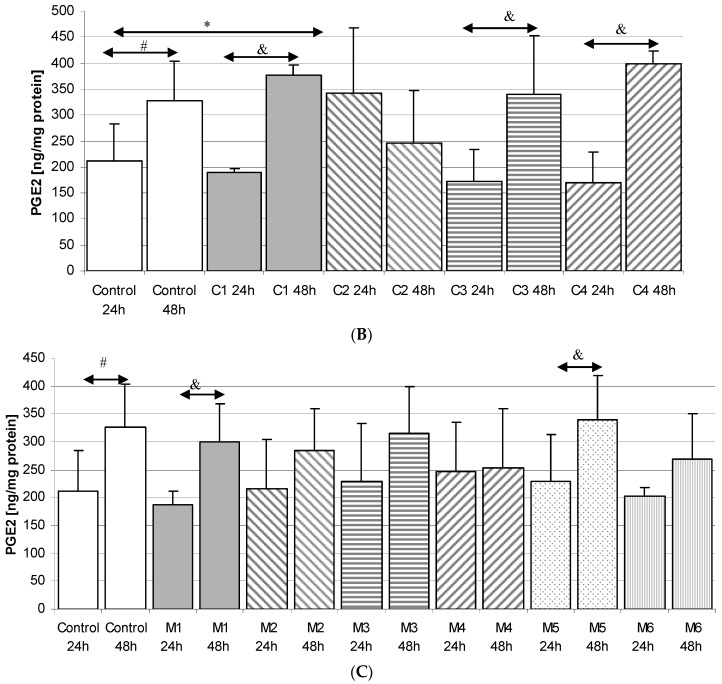
The levels of prostaglandin E_2_ (PGE_2_) in macrophages cultured with various plates and dedicated screws used for the surgical treatment of condylar fractures. Macrophages were cultured for 24 h and 48 h in RPMI medium with 10% FBS with various plates and dedicated screws: (**A**) SYNTES (S1, S2, S3); (**B**) MARTIN (C1, C2, C3, C4); (**C**) MEDARTIS (M1, M2, M3, M4, M5, M6); for details see Table 1). After incubation, the cells were harvested by scraping and PGE_2_ concentration was measured by ELISA. Experiments were conducted as six separate assays, each assay in three replicates, (*n* = 18). * Statistically significant differences in comparison to control 24 h (*p* ≤ 0.05), # Statistically significant differences in comparison to control 48 h (*p* ≤ 0.05), & Statistically significant differences in comparison to appropriate plate 24 h (*p* ≤ 0.05). Control cells were incubated in RPMI medium with 10% FBS.

Incubation of macrophages with the plates resulted in a statistically significant increase in PGE_2_ concentration for the S1 24 h plate compared to S1 48 h (by 80%, *p* = 0.049). Extension of incubation time also increased PGE_2_ concentration for S2 24 h compared to S2 48 h (132%, *p* = 0.035) and S3 24 h compared to S3 48 h (53%, *p* = 0.020). However, PGE_2_ levels in macrophage cultures incubated with SYNTES plates were never statistically significantly higher compared to Control 48 h, Figure 3A.

#### 2.3.2. Incubation of Macrophages with MARTIN Plates (C)

Incubation of cells with MARTIN plates for 48 h resulted in an increase in PGE_2_ concentration in macrophages. Such dependence was shown for C1 24 h as compared to C1 48 h (increase by 100%, *p* = 0.039); C3 24 h vs. C3 48 h (by 97%, *p* = 0.047); C4 24 h vs. C4 48 h (by 136%, *p* = 0.031). However, a statistically significant increase (by 62%, *p* = 0.035) was observed only for macrophages incubated with C2 24 h vs. Control 24 h. Extending the incubation time to 48 h did not result in a further increase in prostaglandin levels and even decreased (however non significantly). There was no statistically significant increase in PGE_2_ concentration in the macrophages incubated under these conditions for 48 h, Figure 3B.

#### 2.3.3. Incubation of Macrophages with MEDARTIS Plates (M)

It was observed that the incubation time significantly affected PGE_2_ concentrations in macrophages incubated with M1 plates for 24 h when compared with M1 48 h (increase by 160%, *p* = 0.005) and M5 24 h vs. M5 48 h (increase by 148%, *p* = 0.031). However, incubation with MEDARTIS plates resulted in no significant increase in PGE_2_ compared to Control 48 h, Figure 3C.

### 2.4. Thromboxane TXB_2_ in THP-1 Monocytes

Incubation time did not significantly affect the concentration of thromboxane in the cell culture under control conditions—TXB_2_ levels were not significantly different in Control 24 h and Control 48 h.

#### 2.4.1. Incubation of THP-1 Cells with SYNTES (S) Plates

**Figure 4:**
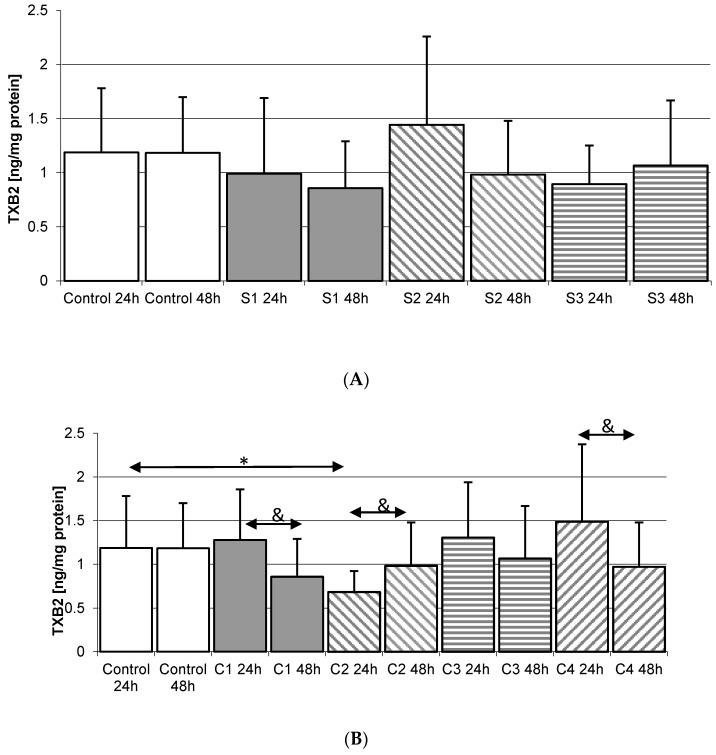

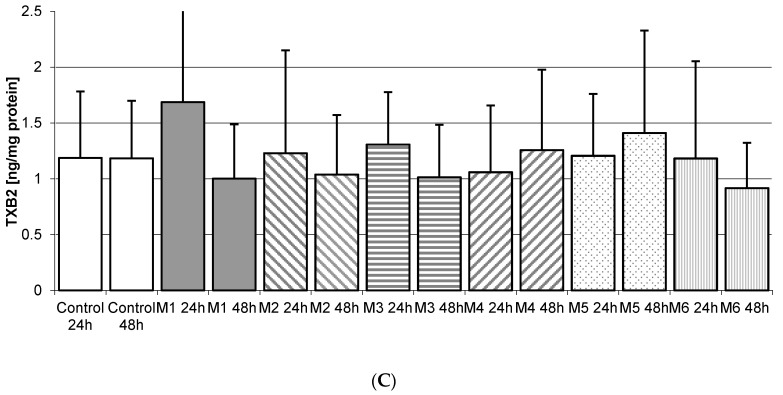
The levels of thromboxane TXB_2_ in THP-1 monocytes cultured with various plates and dedicated screws used for surgical treatment of condylar fractures. THP-1 cells were cultured for 24 h and 48 h in RPMI medium with 10% FBS with various plates and dedicated screws: (**A**) SYNTES (S1, S2, S3); (**B**) MARTIN (C1, C2, C3, C4); (**C**) MEDARTIS (M1, M2, M3, M4, M5, M6); for details see Table 1. After incubation cells were harvested by scraping and TXB_2_ concentration was measured by ELISA method. Experiments were conducted as six separate assays, each assay in three replicates, (*n =* 18). * Statistically significant differences in comparison to control 24 h (*p* ≤ 0.05), # Statistically significant differences in comparison to control 48 h (*p* ≤ 0.05), & Statistically significant differences in comparison to appropriate plate 24 h (*p* ≤ 0.05). Control cells were incubated in RPMI medium with 10% FBS.

Incubation of THP-1 monocytes with MEDICOM plates for 24 h resulted in no significant increase in TXB_2_. Extending the incubation time to 48 h also did not significantly increase the concentration of thromboxane for any of the plates used, Figure 4A.

#### 2.4.2. Incubation of THP1 Cells with MARTIN Plates (C)

Incubation of THP-1 monocytes over 24 h with C2 plates resulted in a statistically significant decrease in TXB_2_ concentration in the medium vs. Control 24 h (42%, *p* = 0.045). A similar relationship was observed for monocyte incubation with C1 24 h plate compared to C1 48 h (32% decrease in TXB_2_, *p* = 0.035). Extension of incubation time also resulted in a decrease in TXB_2_ concentration when incubated with C4 24 h vs. C4 48 h. Incubation with C2 24 h resulted in a statistically significant increase in thromboxane concentration when compared to C2 48 h. However, for any of the MARTIN plates used, TXB_2_ levels did not statistically significantly differ from Controls 24 h and 48 h, Figure 4B.

#### 2.4.3. Incubation of THP1 Cells with MEDARTIS Plates (M)

The concentration of TXB_2_ in cultures of monocytes incubated with MEDARTIS plates was not statistically significantly higher than control. It was also observed that extension of incubation time did not affect the concentration of thromboxane in cultures incubated with any of the plates used, Figure 4C.

### 2.5. Thromboxane TXB_2_ in THP-1 Macrophages

The concentration of TXB_2_ in macrophages under control conditions was significantly higher during 48 h incubation compared to 24 h incubation (by as much as 108%, *p* = 0.002).

#### 2.5.1. Incubation of Macrophages with SYNTES Plates (S)

**Figure 5:**
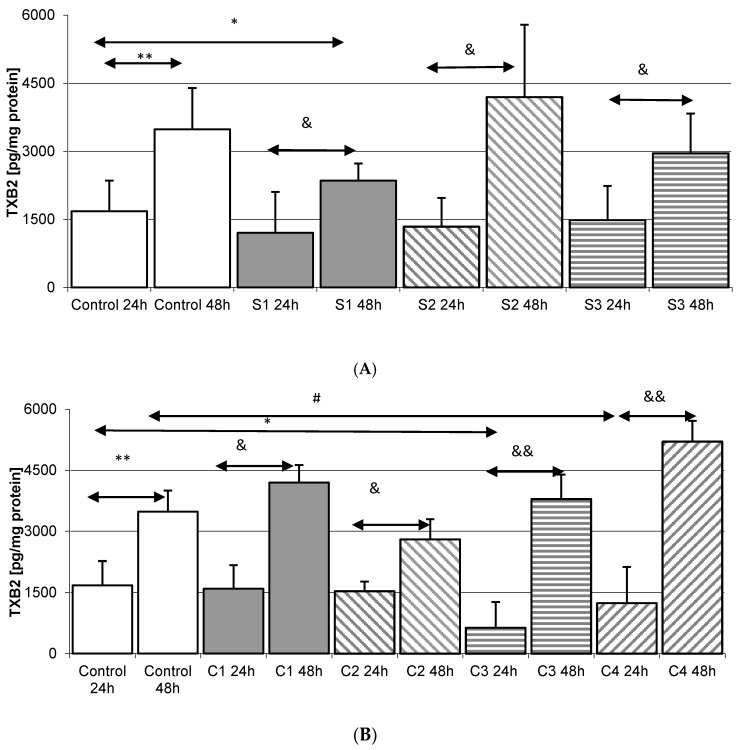

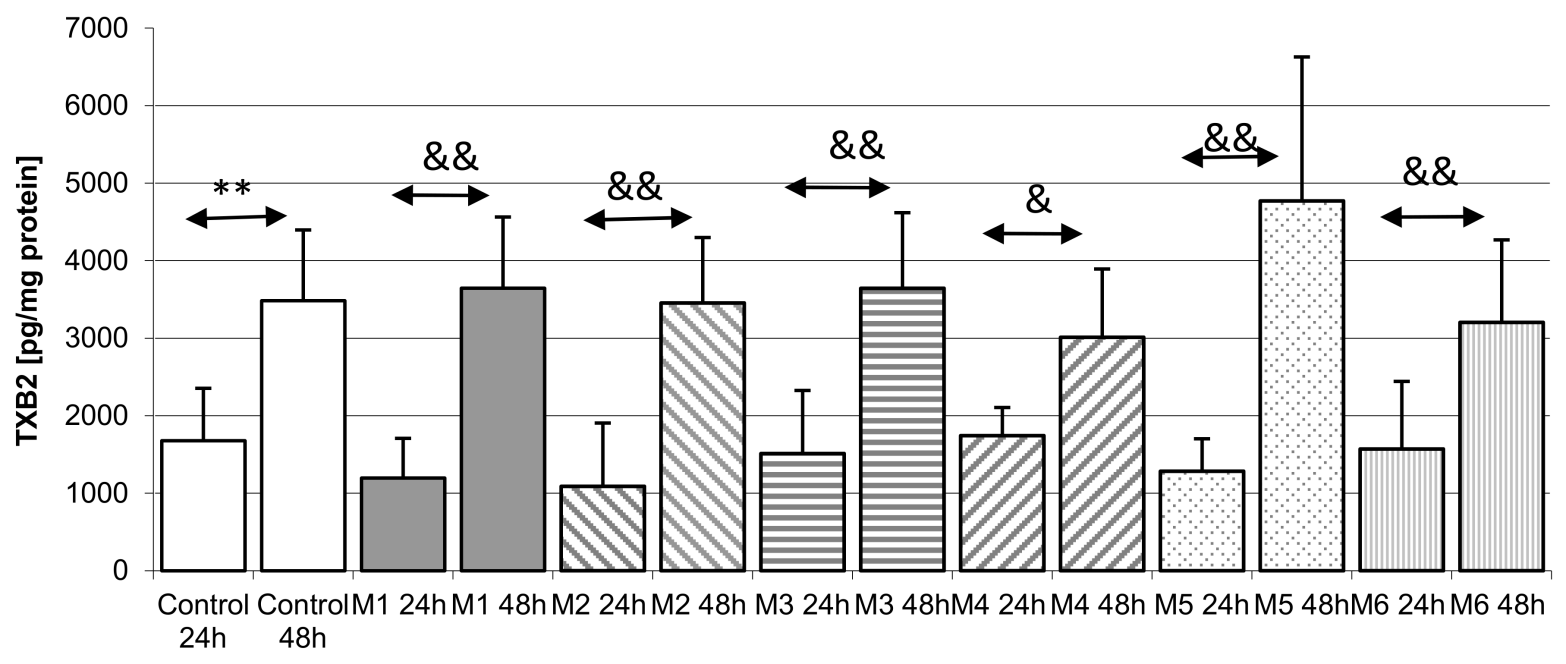
The quantity of thromboxane TXB_2_ in macrophages cultured with various plates and dedicated screws used for surgical treatment of condylar fractures. Macrophages were cultured for 24 h and 48 h in RPMI medium with 10% FBS with various plates and dedicated screws: (**A**) SYNTES (S1, S2, S3); (**B**) MARTIN (C1, C2, C3, C4); (**C**) MEDARTIS (M1, M2, M3, M4, M5, M6); for details see Table 1). After incubation cells were harvested by scraping and TXB_2_ concentration was measured by ELISA. Experiments were conducted as six separate assays, each assay in three replicates, (*n =* 18). * Statistically significant differences in comparison to control 24 h (*p* ≤ 0.05), ** Statistically significant differences in comparison to control 24 h (*p* ≤ 0.001), # Statistically significant differences in comparison to control 48 h (*p* ≤ 0.05), & Statistically significant differences in comparison to appropriate plate 24 h (*p* ≤ 0.05), && Statistically significant differences in comparison to appropriate plate 24 h (*p* ≤ 0.001). Control cells were incubated in RPMI medium with 10% FBS.

Significantly lower TXB_2_ concentrations compared to Control 48 h (by 32%, *p* = 0.045) were found in macrophages incubated with SYNTES S1 plates for 48 h. Incubation of macrophages with S1 24 h plates resulted in a statistically significant increase in TXB_2_ compared to S1 48 h (95%, *p* = 0.029). A significant increase in TXB_2_, by 213% (*p* = 0.010), was also observed when compared with S2 48 h plate. A similar relationship was observed for S3 24 h vs. S3 48 h (significant increase by 99%, *p* = 0.042). The increased in TXB_2_ concentration were never statistically significantly higher than in Control 48 h, Figure 5A.

#### 2.5.2. Incubation of Macrophages with MARTIN Plates (C)

Incubation of macrophages with the C3 24 h plate showed a statistically significant decrease in TXB_2_ concentration (62%, *p* = 0.026) compared to Control 24 h. A significant increase in TXB_2_ in the medium was observed for C1 24 h vs. C1 48 h (by 163%, *p* = 0.025); C2 24 h vs. C2 48 h (82%, *p* = 0.032); C3 24 h vs. C3 48 h (5-fold increase, *p* = 0.002); C4 24 h vs. C4 48 h (4x increase, *p* = 0.0023). In none of these cases was the observed increase significantly different from Control 48, except for C4 48 h (50%, *p* = 0.048), Figure 5B.

#### 2.5.3. Incubation of Macrophages with MEDARTIS Plates (M)

Forty-eight hour long incubation with MEDARTIS plates resulted in a statistically significant increase in medium TXB_2_ levels compared to 24 h incubation; M1 24 h vs. M1 48 h (3-fold increase, *p* = 0.002); M2 24 h vs. M2 48 h (3-fold increase, *p* = 0.0031); for M3 24 h vs. M3 48 h (increase by 140%, *p* = 0.045); M4 24 h vs. M4 48 h (increase by 72%, *p* = 0.035); M5 24 h vs. M5 48 h (nearly 3-fold increase, *p* = 0.001) and M6 24 h vs. M6 48 h (by 103%, *p* = 0.032). There was no statistically significant difference in TXB_2_ concentrations between cultures incubated with MEDARTIS plates and Control 48 h, Figure 5C.

## 3. Discussion

The incubation of THP-1 monocytic cells with a 3D condylar mini-plate and screws did not result in a significant increase in the concentration of TXB_2_ or PGE_2_ in the incubation medium compared to control. It also did not transform THP-1 monocytes into macrophages, as confirmed by the cytometric test (Figure 1). As mentioned, incubation was performed with three different types of titanium 3D implants and dedicated screws from three different manufacturers (Table 1 and Table 2).

Regardless of the manufacturer, none of the elements used in the osteosynthesis of the condylar fractures resulted in a statistically significant increase in PGE_2_ level in macrophages obtained from THP-1 monocytic cells (transformation of monocytes into macrophages was carried out by PMA). The incubation of macrophages obtained from THP-1 cells also did not increase TXB_2_ levels in the incubation medium (Figure 2, Figure 3, Figure 4 and Figure 5). The obtained results suggest that the 3D titanium mini-plates and dedicated screws do not increase the activity of cyclooxygenase-2 (COX-2), and so do not stimulate inflammatory reactions. In addition, differences in the content of implants had no effect on the production of the prostanoids PGE_2_ and TXB_2_ (Table 1 and Figure 2, Figure 3, Figure 4 and Figure 5).

Interestingly, in single cases we did find statistically significantly lower levels of PGE_2_ and TXB_2_ in relation to control, both in the cultures of monocytes and macrophages obtained from THP-1 monocytes [28]. These finding somewhat confirm the suggestions on the anti-inflammatory properties of titanium, which appeared as early as 1980s [28], and were later reinforced by the research in the 1990s when Overgaard et al. proved a less intense inflammatory reaction after the use of titanium implants in the treatment of arthritis compared to polyethylene implants [29].

Our results also indirectly confirm the observations of Chen et al. who show that titanium alloy result in a less intense inflammatory reaction in the culture of macrophages compared to stainless steel; they also report a lower number of macrophages on the surface of the titanium alloy plate compared to a stainless steel plate [30]. Similarly, Olivares-Navarette et al. argue that titanium material used in orthopedic surgeries results in a reduced inflammation, as seen in the lower production of pro-inflammatory cytokines and an increase in the synthesis of the anti-inflammatory cytokine Il-10, produced mainly by monocytes, macrophages, and lymphocytes [31].

Although most reports indicate that titanium surfaces result in a lower migration of monocytes and macrophages and inflammation compared to other materials, there are also papers indicating that titanium alloy implants induce the production of COX-2 [32]. For example, according to Nikura et al. phagocytosis of particles from titanium alloys (Ti-6AL-4V) results in the induction of COX-2, which in turn leads to elevated synthesis and increase in the amount of PGE_2_ in the medium of macrophage cultures obtained from the U937 monocytic cells [33]. In their following experiment, the researchers show that the induction of COX-2 and PGE_2_ synthesis in human macrophages incubated in vitro with titanium particles, significantly depend on the use of cyclic stretch, which additionally induces the production of PGE_2_ [34].

In summary, we would like to emphasize the novel nature of our research, first to examine the effect of titanium 3D plates on the synthesis of PGE_2_ and TXB_2_ in cultures of human monocytes/macrophages. Despite some differences in the content of the implant devices coming from three different manufacturers, our results showed that these plates did not statistically significantly increase the production of these prostanoids (Table 1). The prevention of excessive production of prostanoids can also be attributed to the higher resistance of the 3D condylar plates to forces and tension compared to the previously used plates, as shown by Fujishiro et al. [34].

## 4. Material and Methods

### 4.1. Reagents

THP-1 monocytic cells was obtained from American Type Culture Collection (ATCC, Rockville, MD, USA). RPMI medium, glutamine, and antibiotics (penicillin and streptomycin), phosphate buffered saline (PBS), phorbol 12-myristate 13-acetate (PMA) were purchased from Sigma–Aldrich (Poznań, Poland). Fetal bovine serum was purchased from Gibco (Gibco, Paisley, UK). Prostaglandin E_2_ EIA Kit and Thromboxane B_2_ EIA Kit were purchased from Cayman Chemical (Ann Arbor, MI, USA); Micro BCA Protein Assay kit was purchased from Thermo Scientific (Pierce Biotechnology, Rockford, IL, USA).

### 4.2. 3D Condylar Titanium Plates

Sets consisting of 3D titanium condylar plates and dedicated screws were obtained from three manufacturers. Plates obtained from DePuy Synthes (Renam, MA, USA, manufacturer no. 1) were made of pure titanium TiCP–DIN ISO 5832-2, with screws made of titanium alloy (TAN)–DIN 5832-11. Plates manufactured by KLS Martin (Mühlheim, Germany, manufacturer no. 2) were made of pure titanium in accordance with DIN-ISO 5832-2, DIN 17850 and ASTM F 67. Plate-compatible screws were made of titanium alloy (Ti-6Al-4V) according to DIN ISO 5832-3, DIN 17851 and ASTM F 136a. Plates provided by Medartis (manufacturer no. 3) were made of pure titanium ASTM 67, ISO 5832-2, with screws made of titanium alloy ASTM F136, ISO 5832-2. Detailed manufacturer’s data on plates and screws used for testing are provided in Table 1. Data on 3D titanium condylar plates and dedicated screws, along with the assigned symbols, are presented in Table 2 [35].

### 4.3. Cell Culture and Treatment

The experiments were conducted on human monocytic cell line THP-1 and macrophages derived from THP-1 cells. Cells were cultured in Roswell Park Memorial Institute medium (RPMI) 1640 (Sigma, St. Louis, MO, USA), supplemented with 100 IU/mL penicillin and 10 µg/mL streptomycin (Life Technologies, Inc., Grand Island, NY, USA) in the presence of 10% heat-inactivated fetal bovine serum (FBS, Life Technologies, Grand Island, NY, USA). The cells were cultured in a humidified atmosphere at 37 °C in 5% CO_2_ and the media were refreshed every 48 h. Before the experiment THP-1 cells were plated in culture flasks at an initial density of 2.5 × 105 cells/well (Corning, Cambridge, MA, USA). The differentiation of THP-1 cells into macrophages was achieved by administration 100 nM PMA for 24 h [36].

THP-1 cells model has some advantages over human macrophages isolated from blood of heavy metals-exposed people. Their homogenous genetic background minimizes the degree of variability in the cell phenotype [37,38]. Such cell model eliminates the influence of other environmental factors that may interfere with the examined mechanisms of heavy metals action. Therefore THP-1 cells experimental system represents a convenient model for the studies of molecular mechanisms of heavy metals action on macrophages in relation to inflammatory processes. Such experimental model using THP-1 cells was also used for the studies of inflammatory processes induced by various factors, including heavy metals (such as cadmium) [38], vanadium [23] and other elements (such as fluoride) [39].

### 4.4. Verification of Plate-Induced Activation of THP-1 Monocytes and Initiation of Inflammatory Reaction

In the first experiment THP-1 cells were cultured for 24 h and 48 h in RPMI medium with 10% FBS with 3D condylar plates and screws used for surgical treatment of patients with condylar fractures. Plates and the suitable screws were obtained from three different manufacturers: (1) manufacturer no. 1 (S1, S2, S3); (2) manufacturer no. 2 (C1, C2, C3, C4); (3) manufacturer no. 3 (M1, M2, M3, M4, M5, M6); for details see Table 1. After incubation, the cells were harvested by scraping and the pellets were obtained by centrifugation (800 ×*g*, 10 min). Afterwards, cool PBS was added to the pellets and the samples were stored at −80°C until the further analyses. The measurement of protein concentration was conducted by Micro BCA Protein Assay Kit (Thermo Scientific, Rockford, IL, USA). The remaining supernatants were placed in new tubes and stored at −80 °C until further analyses, i.e., the extraction and measurement of PGE_2_ and TXB_2_ concentrations by ELISA method. Differentiation of THP-1 monocytic cells into THP-1 macrophages (activation of THP-1 monocytic cells) was measured by flow cytometry [40,41].

### 4.5. Flow Cytometry Measurement of THP-1 Cells Differentiation Into Macrophages

The activity of THP-1 monocytic cells (their differentiation into macrophages without PMA but only in the presence of the studied plates) was checked by expression of CD 14 and CD 68 antibodies. The expression of CD14 and CD68 were evaluated by flow cytometry using mouse anti-human CD14 FITC and mouse anti-human CD64 Alexa Fluor 647 clone Y1:82A antibody (BD Pharmingen, San Diego, CA, USA). Cells were compared to an isotype control, mouse IgG_1_ κ and mouse IgG_2b_ κ (BD Pharmingen). Briefly, the cells were stained in phosphate-buffered saline (PBS, Ca^2+^- and Mg^2+^-free) supplemented with 2% bovine calf serum (BCS, Hyclone, Logan, UT, USA). After the final wash, cells were re-suspended in PBS and analyzed by FACS using the Navios flow cytometer (Beckman Coulter, Brea, CA, USA).

### 4.6. Verification of Plate-Induced Initiation of the Inflammatory Reaction in Macrophages

In the second experiment THP-1 macrophages were cultured for 24 h and 48 h in the same condition and with the same plates as in the first experiment. After incubation cells were harvested as previously. Then protein levels were measured by Micro BCA Protein Assay Kit (Thermo Scientific, Rockford, IL, USA); PGE_2_ and TXB_2_ were determined by ELISA.

### 4.7. The Measurements of PGE_2_ Concentration

PGE_2_ was extracted from the cells with the use of Bakerbond SPE columns (J.T. Baker, Phillipsburg, NJ, USA), as described in manufacturer’s instructions. The concentrations of released PGE_2_ were measured spectrophotometrically in the culture supernatants by using the PGE_2_ Enzyme Immunoassay Kit (Cayman Chemical, Ann Arbor, MI, USA) according to the manufacturer’s protocol [40].

### 4.8. The Measurements of TXB_2_ Concentration

TXB_2_ was assayed in the culture supernatants according to the manufacturer’s instructions. SPE method and Bakerbond columns were used for extracting TXA_2_ from the cells. As TXA_2_ has a short half-life (37 s) and is rapidly non-enzymatically hydrolyzed to its stable derivative TXB_2_, the Thromboxane B2 Enzyme Immunoassay Kit (Cayman Chemical, Ann Arbor, MI, USA) was used to indirectly measure free TXA_2_ [41].

### 4.9. Protein Concentration

Protein concentration was measured by a MicroBCA Protein Assay Kit (Thermo Scientific, Pierce Biotechnology, Rockford, IL, USA) using a spectrophotometer (UVM340, ASYS). The Thermo Scientific™ Micro BCA™ Protein Assay Kit is a detergent-compatible bicinchoninic acid formulation for the colorimetric detection and quantitation of total protein. The unique, patented method uses bicinchoninic acid (BCA) as the detection reagent for Cu^+1^, which is formed when Cu^+2^ is reduced by protein in an alkaline environment. A purple-colored reaction product is formed by the chelation of two molecules of BCA with one cuprous ion (Cu^+1^). This water-soluble complex exhibits a strong absorbance at 562 nm that is linear with increasing protein concentrations. The result is an extremely sensitive colorimetric protein assay in a test tube or microplate assay format.

### 4.10. Statistical Analysis

The statistical analysis of obtained results was conducted using Statistica 10 software (Statsoft, Kraków, Poland). The results were expressed as arithmetical mean ± standard deviation (SD). The distribution of variables was evaluated using the Shapiro-Wilk W-test. The nonparametric tests were used for further analyses, because distribution in most cases deviated from normal distribution. The results were analyzed by the Mann-Whitney U test. The level of significance was established at *p* < 0.05.

## 5. Conclusions

Osteosynthesis of condylar fractures using 3D titanium mini-plates seems to be a good alternative to traditional plates, not only due to their desirable technical parameters but also because they do not stimulate the cyclooxygenase-dependent production of prostanoids, thus limiting the development of inflammatory reactions.

## Figures and Tables

**Table 1:** Detailed manufacturer’s data on plates and screws used for testing.

Metal Alloy	Element (Weight)								
	Fe	O	N	C	H	Al	V	Other	Ti
Ti grade 2 (ASTMF 67:2000)	0.3	0.25	0.03	0.08	0.0125				rest
Ti6Al4V ELI grade5 (ASTM F136; ISO5832-3) (TAV)	0.25	0.13	0.05	0.08	0.012	5.5–6.5	3.5–4.5		rest
Ti6Al7Nb (ASTM F1295; ISO:5832-11) (TAN)	0.25	0.20	0.05	0.08	0.009	5.5–6.5		Nb 6.5–7.5 Ta max. 0.5	rest

**Table 2:** 3D Titanium condylar plates and dedicated screws.

Screw/Plate	Manufacturer	Catalog Number	Symbol
Plate	SYNTHES	04.503.834	S1
Plate	SYNTHES	04.503.833	S2
Screw	SYNTHES	04.503.406.01C	S3
Screw	KLS MARTIN	25-872-09	C1
Plate	KLS MARTIN	25-283-05	C2
Plate	KLS MARTIN	25-285-05	C3
Screw	KLS MARTIN	25-882-09	C4
Screw	MEDARTIS	M-5240.06	M1
Screw	MEDARTIS	M-5245.06	M2
Plate	MEDARTIS	M-4318	M3
Plate	MEDARTIS	M-4852	M4
Plate	MEDARTIS	M-4894	M5
Plate	MEDARTIS	M-4658	M6
